# Role of Alpha-Defensins 3 and 5 in Diabetic Complications: Associations with Nephropathy and Metabolic Parameters in Type 2 Diabetes

**DOI:** 10.3390/metabo16080570

**Published:** 2026-08-11

**Authors:** Yuliyan Naydenov, Vera Karamfilova, Yavor Assyov, Diana Nikolova, Savelia Yordanova, Zdravko Kamenov, Boris Bogov, Julieta Hristova, Antoaneta Gateva

**Affiliations:** 1 Department of Internal Medicine, Faculty of Medicine, Medical University of Sofia, 1431 Sofia, Bulgaria; dr.ynaydenov@gmail.com (Y.N.); vkaramfilova@mail.bg (V.K.); yavovian@abv.bg (Y.A.); nikolowa.diana@abv.bg (D.N.); savi_gandeva@abv.bg (S.Y.); zkamenov@hotmail.com (Z.K.); bbogov@yahoo.com (B.B.); 2 Department of Nephrology, Internal Disease Clinic, University Hospital “Saint Anna”, 1750 Sofia, Bulgaria; 3 Department of Clinical Laboratory, Faculty of Medicine, Medical University of Sofia, 1431 Sofia, Bulgaria; julieta_sd@yahoo.com

**Keywords:** DEFA3, DEFA5, alpha-defensins, type 2 diabetes, diabetic nephropathy, innate immunity, inflammation

## Abstract

**Background/Objectives:** DEFA3 and DEFA5 are alpha-defensins, which are small cationic antimicrobial peptides that are part of the innate immune system. DEFA3 encodes human neutrophil peptide-3 (HNP-3), primarily expressed in neutrophils, while DEFA5 encodes human defensin-5 (HD-5), predominantly expressed in Paneth cells of the small intestine. Both defensins show elevated levels in diabetes and are strongly associated with diabetic nephropathy, with the highest concentrations found in patients with this complication. We evaluated serum levels of DEFA3 and DEFA5 in 160 participants (93 patients with T2DM and 67 controls without carbohydrate disturbances) and their associations with diabetic nephropathy, as well as peripheral and cardiac autonomic neuropathy and anthropometric and metabolic parameters. **Methods:** This was a monocentric, cross-sectional, observational study conducted at the Endocrinology and Metabolic Disorders Clinic of Alexandrovska Hospital in Sofia. Main methods included detailed clinical and anthropometric assessments, diagnosis of peripheral neuropathy via the Neuropathy Disability Score (NDS), evaluation of cardiac autonomic neuropathy using heart rate variability analysis and Ewing cardiovascular reflex tests, comprehensive laboratory investigations with fasting blood samples, and measurement of serum DEFA3 and DEFA5 levels by ELISA kits. **Results:** Serum DEFA3 and DEFA5 levels did not differ significantly between patients with T2DM and controls without carbohydrate disturbances, nor by sex or menopausal status. Patients with diabetic nephropathy showed significantly higher levels of both DEFA3 (345.7 ± 77.9 pg/mL vs. 299.2 ± 100.3 pg/mL; *p* = 0.042) and DEFA5 (5.3 ± 10.1 pg/mL vs. 2.9 ± 2.9 pg/mL; *p* = 0.044). DEFA5 levels were also elevated in participants with increased albumin-to-creatinine ratio (4.9 ± 9.9 pg/mL vs. 2.2 ± 1.5 pg/mL; *p* = 0.043). No significant differences were observed in patients with peripheral neuropathy, cardiac autonomic neuropathy, retinopathy, or macroangiopathy, although a non-significant trend toward higher values was noted. DEFA3 demonstrated moderate discriminatory ability for diabetic nephropathy (AUC = 0.641; *p* = 0.037), superior to DEFA5 (AUC = 0.570; *p* = 0.303). DEFA3 showed multiple weak positive correlations with diabetes duration, body weight, BMI, total and LDL-cholesterol, serum creatinine, and uric acid, while DEFA5 correlated only with total cholesterol. **Conclusions:** Circulating DEFA3 and DEFA5 are associated with diabetic nephropathy and selected metabolic and renal parameters in patients with type 2 diabetes, with DEFA3 showing moderate discriminatory capacity. The associations were largely attenuated after covariate adjustment, suggesting that these alpha-defensins likely reflect downstream innate immune activation and renal stress rather than acting as independent drivers of complications.

## 1. Introduction

The global prevalence of type 2 diabetes is 11.11% (affecting 589 million adults aged 20–79 years in 2024), projected to rise to 12.96% (853 million people) by 2050 [1]. Type 2 diabetes accounts for over 96% of all diabetes cases worldwide [2]. Diabetic complications affect the majority of patients with diabetes, with substantial global burden. The TODAY study demonstrated that the cumulative incidence of any microvascular complication reached 50% by 9 years and 80.1% by 15 years after diagnosis [3]. Specifically, kidney disease developed in 54.8%, nerve disease in 32.4%, and eye disease in 51.0% of participants by 15 years. Macrovascular complications, including coronary artery disease (8.2%), heart failure (3.3%), and stroke (2.2%), also impose substantial burden [4]. Diabetic nephropathy (diabetic kidney disease, DKD) is a chronic kidney disease (CKD), characterized by specific pathologic structural and functional changes in the kidneys that lead to increased urinary albumin excretion and progressive decline in glomerular filtration rate (GFR) [5,6]. It is the leading cause of end-stage renal disease in most developed countries and affects approximately 20–40% of patients with diabetes. DKD is defined as persistent eGFR less than 60 mL/min/1.73 m^2^, albuminuria with an albumin-to-creatinine ratio (ACR) of 30 mg/g or greater, or other markers of kidney damage persisting for at least 3 months in patients with diabetes [7,8]. Normal to mildly increased albuminuria is defined as less than 30 mg/g creatinine, moderately elevated albuminuria as 30 to less than 300 mg/g creatinine, and severely elevated albuminuria as 300 mg/g creatinine or greater.

Diabetic peripheral neuropathy (DPN) affects approximately 20–50% of people with diabetes, with prevalence varying by diabetes type, duration, and diagnostic criteria used. The incidence is 8.8 cases per 1000 person-years in type 1 diabetes and 24–27 cases per 1000 person-years in type 2 diabetes [9,10,11,12]. DAN (diabetic autonomic neuropathy) includes cardiovascular autonomic neuropathy (CAN), gastrointestinal manifestations, genitourinary dysfunction, sudomotor dysfunction, hypoglycemia unawareness, impaired neurovascular function and pupillary abnormalities.

DEFA3 and DEFA5 are alpha-defensins, which are small cationic antimicrobial peptides of the innate immune system with 29–35 amino acid residues and six invariant cysteines forming intramolecular disulfide bonds. DEFA3 encodes human neutrophil peptide-3 (HNP-3) on chromosome 8p23, which is primarily expressed in neutrophils, where it constitutes more than 5% of total cellular protein. HNP-3 is also stored in neutrophil granules and released during immune activation. Other functions include broad-spectrum antimicrobial activity against bacteria, fungi, and some enveloped viruses, as well as immune modulation [7,13]. DEFA5 encodes human defensin-5 (HD-5), which is located on chromosome 8p23, positioned telomerically to the myeloid defensin gene cluster that includes DEFA3 [14]. It is predominantly expressed in Paneth cells of the small intestinal crypts, where it is stored in secretory granules in precursor form and processed by trypsin upon secretion [15]. HD-5 is also constitutively expressed throughout the urinary tract, including the kidney (particularly distal nephron and collecting tubules), ureter, and bladder urothelium [7,16]. Similarly to HNP-3, HD-5 exerts potent antimicrobial activity through membrane disruption but also plays a critical role in maintaining intestinal homeostasis by helping shape the composition of colonizing microbiota, and its reduced expression is a fundamental feature of ileal Crohn’s disease [17,18]. Beyond direct antimicrobial killing, both HNP-3 and HD-5 share immunomodulatory functions including chemotaxis of macrophages, T lymphocytes, and mast cells through a common receptor using Gαi proteins and MAPK signaling, and they can both promote and suppress inflammatory responses depending on the cellular context [19,20].

Serum alpha-defensin concentrations (DEFA1, DEFA2, and DEFA3) are significantly elevated in diabetic patients with macroalbuminuria compared to those with microalbuminuria or normoalbuminuria, with the highest concentrations found in diabetic patients with nephropathy (49.4 ± 4.8 ng/mL) compared to those without complications, as demonstrated in the FinnDiane Study [7,13]. Alpha-defensin levels correlate with systolic blood pressure, HDL-cholesterol, total cholesterol, age, and estimated glomerular filtration rate, and serve as a clinical risk marker for cardiovascular disease-related morbidity and mortality in type 1 diabetes with a hazard ratio of 2.8 for the upper versus lower tertile [13,14]. While DEFA5 is constitutively expressed throughout the urinary tract, including the distal nephron and collecting tubules, and increases with pyelonephritis, its specific role in diabetic nephropathy remains largely unexplored, with most research focusing on its antimicrobial function in urinary tract infections rather than diabetic kidney disease [21].

The present study aimed to evaluate serum concentrations of DEFA3 and DEFA5 in patients with type 2 diabetes mellitus (T2DM) compared to individuals without carbohydrate disturbances and to investigate their associations with diabetic nephropathy, peripheral neuropathy, cardiac autonomic neuropathy, and key anthropometric and metabolic parameters.

## 2. Materials and Methods

### 2.1. Study Design

This was a monocentric, cross-sectional, observational study conducted at the Endocrinology and Metabolic Disorders Clinic of Alexandrovska Hospital, Sofia. Patients were enrolled between August 2023 and September 2025. Eligible participants were between 18 and 65 years of age, had confirmed diagnosis of T2DM with duration between 2 and 20 years, and were able to provide informed consent. Individuals were excluded if they had coexisting neurological diseases unrelated to diabetes, acute complications of diabetes mellitus (diabetic ketoacidosis, hyperosmolar hyperglycemic state, hyperosmolar hyperglycemic coma, hypoglycemic coma), type 1 diabetes mellitus (T1DM), diagnosed neoplasm or heart failure (NYHA class III–IV), chronic inflammation, occult infections, and autoimmune diseases. The study was approved by the Ethics Committee of Scientific Research at Medical University–Sofia (KENIMUS). All included patients signed informed consent. The study was conducted in accordance with the Declaration of Helsinki and approved by the Ethics Committee of the Medical University Sofia—(Protocol No. 11/11 July 2023).

### 2.2. Clinical and Anthropometric Assessment

Detailed medical history was obtained, including diabetes duration, current medication, comorbidities, and complications. Anthropometric measurements included height (cm), body weight (kg), body mass index (BMI, kg/m^2^), waist circumference (measured midway between the lower rib margin and iliac crest at the mid-axillary line), hip circumference (at the level of the greater trochanter), waist-to-hip ratio (WHR), and waist-to-stature ratio (WSR) (Table 1).

### 2.3. Diagnosis of Nephropathy

Diagnosis of diabetic nephropathy was made using the following criteria—persistent eGFR less than 60 mL/min/1.73 m^2^ and/or albuminuria with ACR 30 mg/g or greater; or other markers of kidney damage persisting for at least 3 months in patients with diabetes.

### 2.4. Diagnosis of Neuropathy

#### 2.4.1. Clinical Neuropathy Assessment

Peripheral neuropathy was assessed by an Neuropathy Disability Score (NDS) using a 10 g monofilament for tactile sensitivity (≥4 points per foot; absence at >2 sites scores 1 point), 128 Hz Rydel–Seiffer tuning fork for vibration perception, thermal discriminator for temperature sensation, and reflex hammer for ankle reflexes. Each sensory modality (vibration, temperature, and pinprick) was scored as 0 = present (normal) or 1 = absent/reduced for each foot (maximum 2 points per modality). Ankle reflexes were graded as 0 = normal, 1 = present with reinforcement, or 2 = absent for each side. The total NDS ranged from 0 to 10 points, with higher scores indicating greater neurological impairment. In accordance with previously published thresholds, an NDS > 5 was considered indicative of clinically significant peripheral neuropathy. Higher scores indicated greater neuropathic impairment.

#### 2.4.2. Autonomic Neuropathy

Cardiac autonomic neuropathy was evaluated using the Cardiosys Extra system (MDE GmbH, Heidelberg, Germany) under standardized conditions (morning, fasting ≥ 12 h, no caffeine/alcohol/medications affecting cardiovascular system, 15–20 min rest). Heart rate variability (HRV) was analyzed in time-domain (SDNN, RMSSD, pNN50, HRVi) and frequency-domain (LF, HF, LF/HF) parameters. Twelve-channel ECG with the following spectral analyses (variability of heart rate, QT-interval and QT-depression) was monitored and registered. Ewing cardiovascular reflex tests were performed and scored according to Bellavere criteria (0–2 points per test; total ≥5 points = severe autonomic neuropathy; ≥2 abnormal tests = confirmed cardiac autonomic neuropathy per Toronto Diabetic Neuropathy Expert Group).

In the frequency domain, the high-frequency component (HF, 0.15–0.40 Hz) is synchronous with respiration and primarily reflects vagal (parasympathetic) tone. The low-frequency component (LF, <0.04–0.15 Hz) is associated with changes in vasomotor tone and reflects a combination of sympathetic and vagal influences. The LF/HF ratio represents the balance between low- and high-frequency components, with an increase indicating sympathetic predominance.

### 2.5. Laboratory Investigations

Fasting venous blood samples were collected after ≥12 h overnight fasting. Analyses included complete blood count, HbA1c, fasting glucose, lipid profile (total cholesterol, LDL, HDL, VLDL, triglycerides), liver enzymes (AST, ALT, GGT), creatinine, eGFR, uric acid, total protein, albumin, and urine albumin-to-creatinine ratio (Table 2). All tests were performed in the Central Clinical Laboratory of Alexandrovska Hospital (reference laboratory for Bulgaria).

### 2.6. Cardiovascular Risk Factors

Arterial hypertension was defined as blood pressure ≥140/90 mmHg and/or the use of antihypertensive treatment. Dyslipidemia was defined as total cholesterol >5.2 mmol/L and/or HDL-cholesterol <1.3 mmol/L in women or <1.0 mmol/L in men and/or triglycerides >1.7 mmol/L and/or the use of lipid-lowering treatment. Metabolic syndrome was diagnosed according to the 2009 joint criteria of the International Diabetes Federation (IDF) and the American Heart Association/National Heart, Lung, and Blood Institute (AHA/NHLBI) when at least 3 out of the following 5 risk factors were present: increased waist circumference (>80 cm in women and >94 cm in men), elevated triglycerides (≥1.7 mmol/L), reduced HDL-cholesterol (<1.3 mmol/L in women and <1.0 mmol/L in men), elevated blood pressure (≥130/85 mmHg), and elevated fasting plasma glucose (≥5.6 mmol/L). The number of fulfilled criteria was also recorded for each patient.

### 2.7. Measurement of DEFA3 and DEFA5

Serum levels of DEFA3 and DEFA5 were quantified using commercially available enzyme-linked immunosorbent assay (ELISA) kits according to the manufacturer protocols.

### 2.8. Statistical Analysis

Statistical analysis was performed using IBM SPSS Statistics version 25. Data were checked for missing values, outliers, and anomalies prior to analysis. Descriptive, variation, and graphical analyses were applied. Normality of distribution was assessed using the Kolmogorov–Smirnov and Shapiro–Wilk tests, parametric methods (ANOVA and *t*-test), and non-parametric methods (Mann–Whitney and Chi-square). Descriptive analysis was employed with presentation of frequency distributions in tabular form by study groups. Variation analysis was used for calculating measures of central tendency and dispersion; furthermore, graphical visualization of the results was performed. In cases where normal distribution was not achieved even after transformation, non-parametric tests were used. In addition, one-way analysis of variance (ANOVA) for comparing multiple independent samples, Student’s *t*-test for comparing two independent samples, Mann–Whitney U test for non-parametric comparison of two independent samples, Chi-square test, Spearman correlation analysis to evaluate linear relationships between quantitative variables, binary and multiple logistic regression to quantify the influence of the studied factors, and receiver operating characteristic (ROC) curve analysis to determine optimal cut-off values of quantitative variables for classification of specific conditions were performed. A *p*-value < 0.05 was considered statistically significant, with correction for multiple comparisons performed using the Benjamini–Hochberg false discovery rate (FDR) method.

## 3. Results

### 3.1. Participants

The study included 160 participants (mean age 55.8 ± 8.7 years): 93 patients with T2DM and 67 controls. Patients with T2DM were older and had significantly higher body weight, BMI, waist circumference, WHR, and WSR (*p* < 0.05; Table 3). Mean HbA1c in the diabetes group was 7.79 ± 1.47% and fasting glucose was 7.03 ± 1.61 mmol/L.

Patients with type 2 diabetes were older and had higher body weight, as well as higher waist circumference, WHR, and WSR (indicators of visceral adiposity) compared to participants without carbohydrate disturbances (*p* < 0.05). At the time of the study, the mean glycated hemoglobin (HbA1c) level in patients with diabetes was 7.79 ± 1.47%, while the mean fasting blood glucose was 7.03 ± 1.61 mmol/L.

Peripheral diabetic neuropathy was diagnosed in 72% of the patients with diabetes, diabetic nephropathy in 23.7%, cardiac autonomic neuropathy in 65.8%, diabetic retinopathy in 14%, and coronary artery disease in 18.7%. Additionally, 10.9% of the patients had a history of acute myocardial infarction, 5.5% had a history of stroke, and 5.5% had peripheral arterial disease. Serum levels of DEFA3 and DEFA5 did not differ significantly between patients with T2DM and controls without carbohydrate disturbances, nor according to sex or menopausal status.

**Figure 1:**
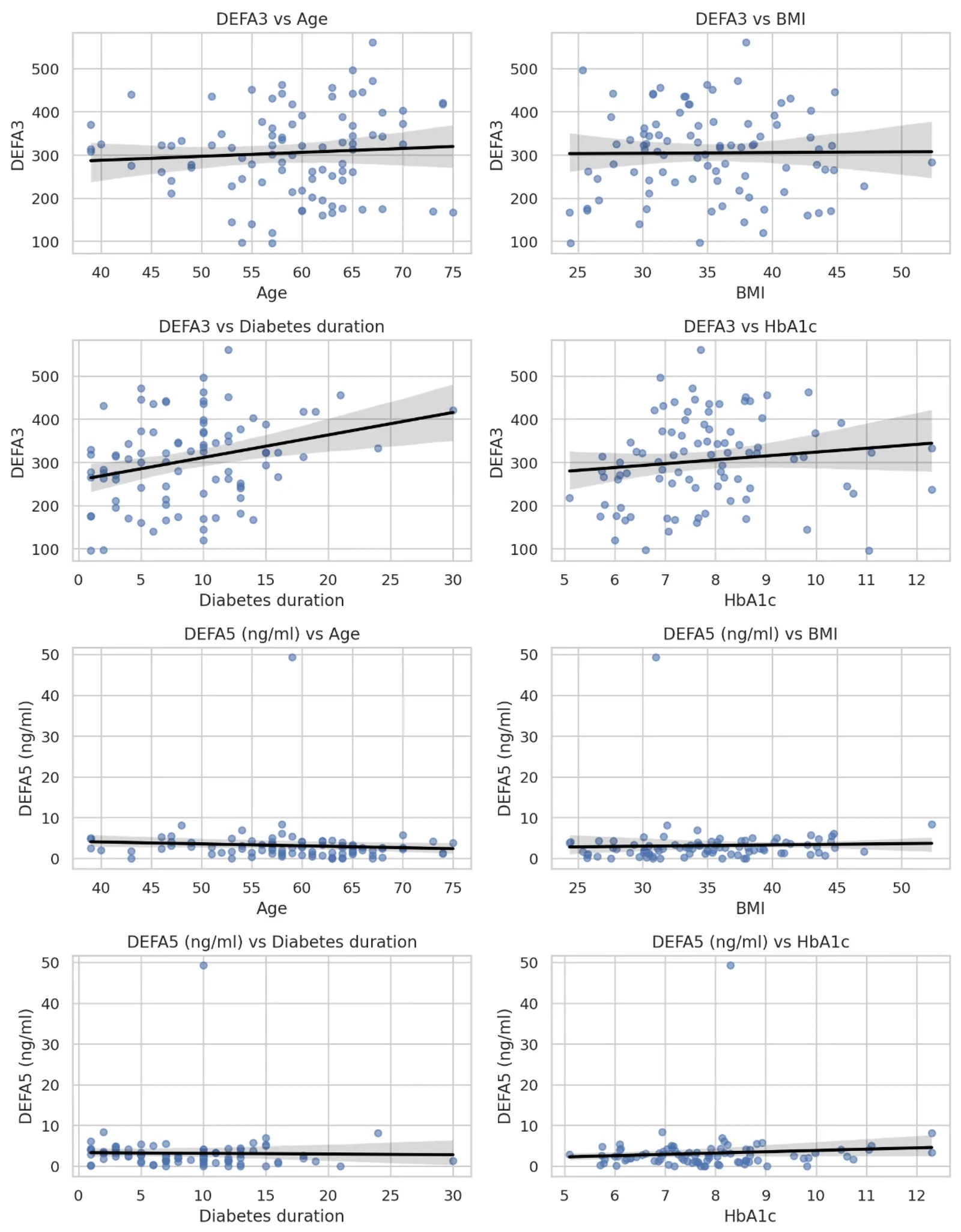
Association of DEFA3 and DEFA5 with various biochemical and metabolic markers.

DEFA3 showed weak positive correlations with diabetes duration, body weight, BMI, total and LDL-cholesterol, serum creatinine, and uric acid, as well as a negative correlation with eGFR. In contrast, DEFA5 correlated significantly only with total cholesterol (Table 3). These associations are visualized in Figure 1.

### 3.2. Diabetic Complications

**Figure 2:**
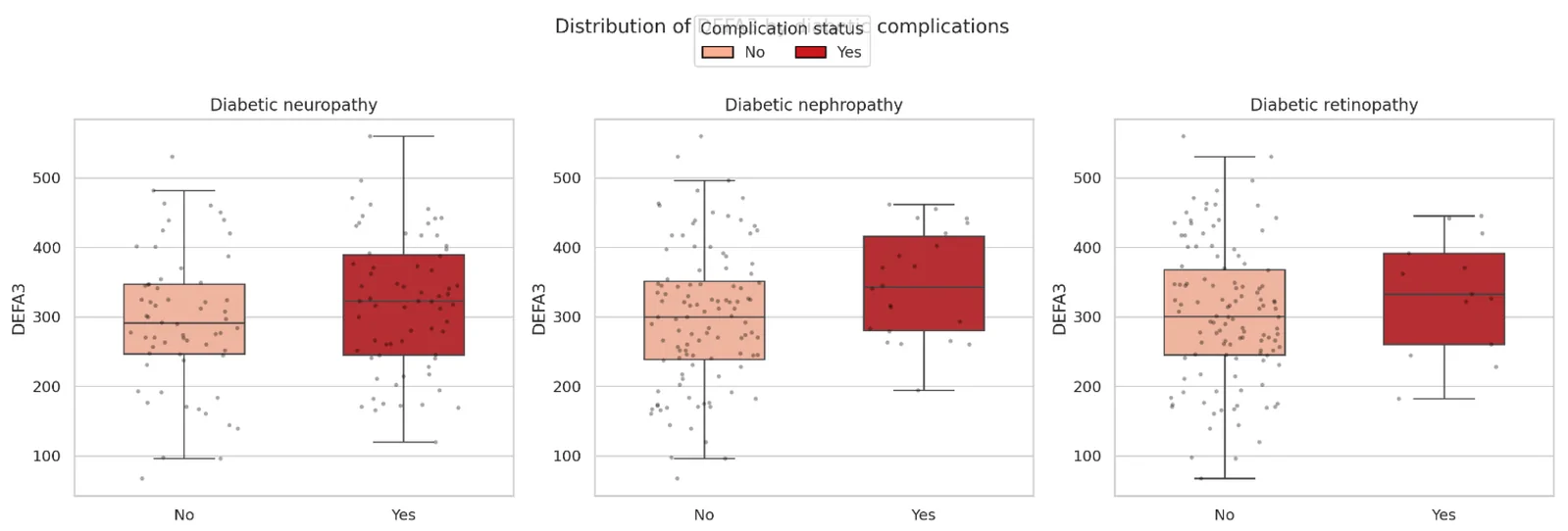
Distribution of DEFA3 by diabetic complications.

**Figure 3:**
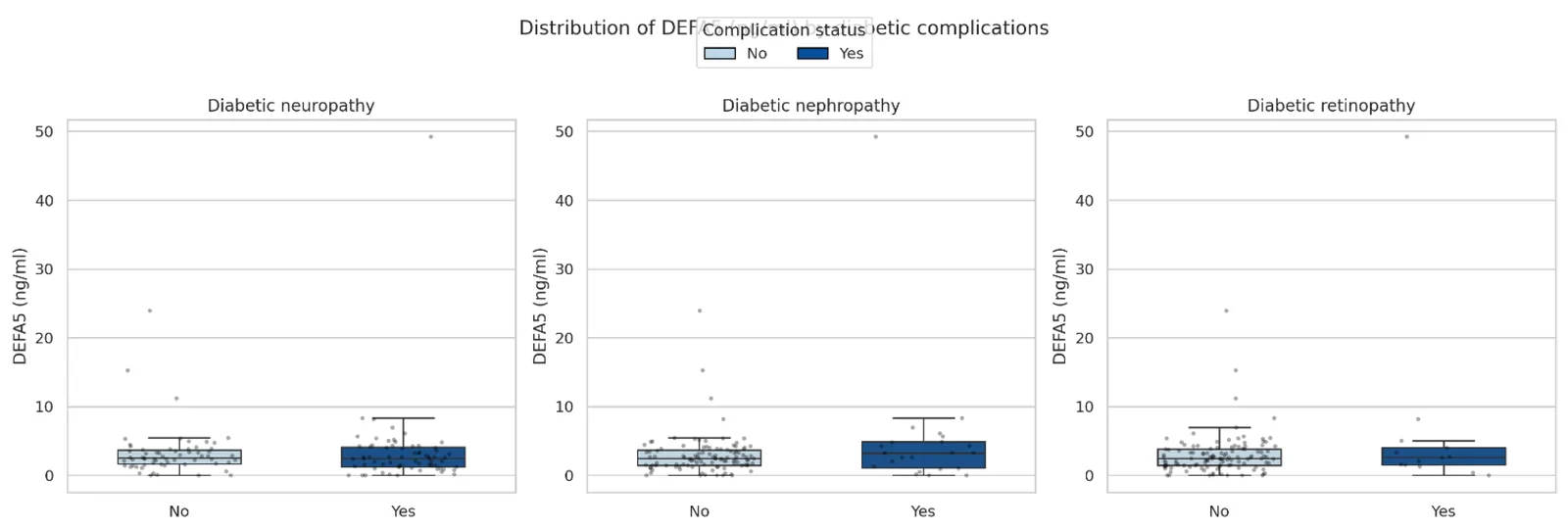
Distribution of DEFA5 (ng/mL) by diabetic complications.

Patients with diabetic nephropathy had significantly higher levels of DEFA3 (345.7 ± 77.9 pg/mL vs. 299.2 ± 100.3 pg/mL; *p* = 0.042) and DEFA5 (5.3 ± 10.1 ng/mL vs. 2.9 ± 2.9 ng/mL; *p* = 0.044). No significant differences were found between patients with and without other diabetic complications—peripheral and autonomic neuropathy, diabetic retinopathy, and diabetic macroangiopathy—although there was a non-significant trend toward higher values in patients with complications. Elevated DEFA5 levels were also observed in patients with increased ACR compared to those with normal levels (4.9 ± 9.9 pg/mL vs. 2.2 ± 1.5 pg/mL; *p* = 0.043) (Figure 2 and Figure 3).

**Figure 4:**
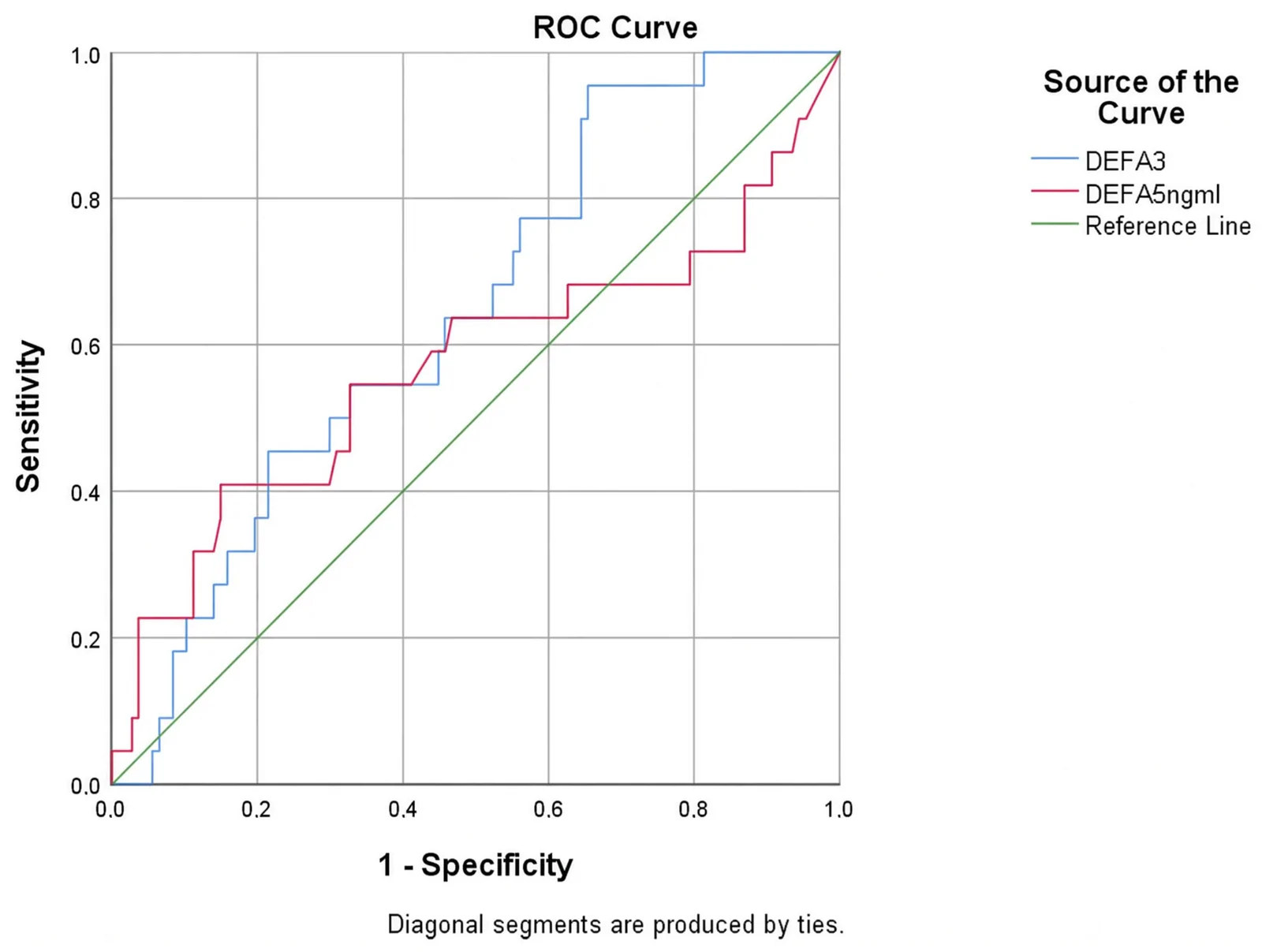
Discriminatory ability of DEFA3 and DEFA5 for the presence of diabetic nephropathy.

DEFA3 demonstrated significantly better discriminatory value than DEFA5 for distinguishing patients with diabetic nephropathy (Figure 4 and Table 4).

In unadjusted logistic regression, DEFA3 showed nominal positive associations with both diabetic neuropathy and diabetic nephropathy. Per 1 SD higher DEFA3, both the odds of diabetic neuropathy (OR 1.80, 95% CI 1.07–3.02; *p* = 0.027) and diabetic nephropathy were higher (OR 1.80, 95% CI 1.06–3.04; *p* = 0.029). DEFA3 was not significantly associated with diabetic retinopathy (OR 1.41, 95% CI 0.77–2.58; *p* = 0.268) or autonomic neuropathy (OR 1.06, 95% CI 0.65–1.73; *p* = 0.826). For DEFA5 (ng/mL), unadjusted analyses did not demonstrate statistically significant associations with complications. Univariable ORs per 1 SD higher DEFA5 were 1.49 for diabetic neuropathy (95% CI 0.45–4.90; *p* = 0.510), 2.88 for diabetic nephropathy (95% CI 0.73–11.33; *p* = 0.129), 1.70 for diabetic retinopathy (95% CI 0.73–3.99; *p* = 0.219), and 0.59 for autonomic neuropathy (95% CI 0.20–1.75; *p* = 0.338).

We next evaluated whether circulating DEFA3 and DEFA5 were independently associated with prevalent diabetes complications in the diabetes-only cohort. Adjusted associations between DEFA3 and DEFA5 and diabetes-related complications were evaluated using multivariable logistic regression models restricted to participants with diabetes (diabetics-only), because diabetes-specific covariates (particularly diabetes duration and HbA1c) are not available/meaningful for non-diabetic participants and can induce complete-case issues and model separation when modeling diabetes status itself. Effects are reported as odds ratios (ORs) per 1 standard deviation (SD) increase in each biomarker. Multiple testing across the four complications was controlled within each biomarker using the Benjamini–Hochberg false discovery rate (BH-FDR), reported as q-values.

For each outcome, we fit three specifications: a model with DEFA3, a model with DEFA5, and a joint model including DEFA3 + DEFA5 simultaneously (each time retaining the same adjustment set). Results are presented as adjusted odds ratios (ORs) with 95% confidence intervals (CIs).

Among participants with complete data for this model, DEFA3 was not associated with neuropathy, with ORs very close to null both when modeled alone and jointly with DEFA5 (DEFA3-only adjusted OR 1.002; 95% CI 0.995–1.008; *p* = 0.608; joint-model OR 1.001; 95% CI 0.995–1.008; *p* = 0.647). DEFA5 also showed no clear association (DEFA5-only adjusted OR 1.204; 95% CI 0.847–1.711; *p* = 0.300; joint-model OR 1.201; 95% CI 0.844–1.710; *p* = 0.308). In other words, after accounting for diabetes duration and renal markers, neither defensin provided evidence of an independent relationship with neuropathy in this dataset.

For nephropathy, DEFA5 showed the strongest (though still borderline) signal in the analysis. In the DEFA5-only adjusted model, the OR was 1.315 (95% CI 0.946–1.826; *p* = 0.103), and in the joint DEFA3 + DEFA5 model, the estimated association for DEFA5 increased slightly to OR 1.373 (95% CI 0.976–1.932; *p* = 0.068). While this does not cross conventional statistical significance thresholds, the point estimate and CI pattern are compatible with a potentially meaningful positive association that the current sample may be underpowered to confirm. By contrast, DEFA3 did not show clear evidence of association with nephropathy (DEFA3-only adjusted OR 1.004; 95% CI 0.997–1.011; *p* = 0.240; joint-model OR 1.006; 95% CI 0.998–1.014; *p* = 0.155). We performed sensitivity analyses for DEFA3 and DEFA5 while including the treatment-related variables available in our dataset, namely antihypertensive treatment, dyslipidemia treatment, SGLT2/GLP-1-based therapy, and other glucose-lowering therapies. For DEFA3, the association with diabetic nephropathy remained significant after medication adjustment (OR 3.13, 95% CI 1.10 to 8.86, *p* = 0.032), suggesting that the observed relationship is not fully explained by the available treatment variables. For DEFA5, the association with diabetic nephropathy was attenuated after medication adjustment (base model OR 15.73, *p* = 0.029; medication-adjusted OR 9.36, *p* = 0.083), indicating that treatment-related confounding may partly contribute. No robust associations were observed for diabetic neuropathy, autonomic neuropathy, or retinopathy for either marker after adjustment.

## 4. Discussion

The present study provides new insights into the role of circulating alpha-defensins DEFA3 (HNP-3) and DEFA5 (HD-5) as markers of innate immune activation in type 2 diabetes mellitus (T2DM) and its microvascular complications. In this monocentric cross-sectional cohort of 160 participants, serum levels of DEFA3 and DEFA5 did not differ significantly between patients with T2DM and non-diabetic controls, nor by sex or menopausal status. However, both defensins were significantly elevated in patients with diabetic nephropathy, with DEFA3 showing superior discriminatory capacity compared with DEFA5. DEFA3 also exhibited broader and stronger correlations with diabetes duration, anthropometric indices (body weight and BMI), dyslipidemia (total and LDL-cholesterol), and renal function parameters (serum creatinine, uric acid, and eGFR). In unadjusted analyses, DEFA3 was nominally associated with both diabetic nephropathy and neuropathy. Adjusted associations between DEFA3 and DEFA5 and diabetes-related complications were evaluated using multivariable logistic regression models restricted to participants with diabetes (diabetics-only) for diabetes duration, HbA1c, and renal markers. Covariates included age, sex, BMI, HbA1c, and diabetes duration. Effects are reported as odds ratios (ORs) per 1 standard deviation (SD) increase in each biomarker. Multiple testing was controlled using the Benjamini–Hochberg false discovery rate (BH-FDR). Neither biomarker retained independent associations, although DEFA5 showed a borderline positive signal for nephropathy (OR 1.373; *p* = 0.068 in the joint model).

These findings align with and extend prior evidence that alpha-defensins are upregulated in the context of diabetic kidney disease.

Our results are consistent with established observations that circulating levels of alpha-defensins (particularly HNP1-3, which includes DEFA3) are markedly elevated in diabetic patients with nephropathy. Saraheimo et al. previously demonstrated increased plasma alpha-defensin (-1, -2, and -3) concentrations in type 1 diabetic patients with nephropathy, linking these elevations to low-grade inflammation and altered lipid profiles [22]. Similarly, Németh et al. reported significantly higher HNP1-3 levels in both type 1 and type 2 diabetes, with the highest concentrations observed in patients with nephropathy (approximately 49.4 ng/mL), followed by neuropathy and cardiovascular complications, independent of diabetes type [23]. More recent data in type 2 diabetes have further shown that alpha-defensins correlate positively with advanced glycation end-products (AGEs), fasting blood glucose, BMI, and diabetes duration, with the strongest elevations in patients with nephropathy compared to other complications [13,14]. The present study corroborates these patterns while providing novel granularity: DEFA3 (neutrophil-derived) appears more sensitive to the systemic inflammatory and metabolic burden of T2DM, whereas DEFA5 (primarily enteric) shows more restricted associations, mainly with total cholesterol and a borderline independent signal for nephropathy after adjustment.

From a pathophysiological perspective, the observed elevations in DEFA3 and DEFA5 likely reflect neutrophil hyperactivation and degranulation, which are well-documented features of the chronic low-grade inflammation that drives diabetic complications. DEFA3, a major component of neutrophil azurophilic granules, is released during NETosis and direct degranulation; in the diabetic milieu, hyperglycemia, oxidative stress, and AGEs promote neutrophil priming and excessive NET formation, which contribute to endothelial injury and glomerular damage in diabetic nephropathy. Experimental and clinical evidence supports this mechanism: neutrophil extracellular traps (NETs) have been directly implicated in glomerular endothelial cell injury and progression of diabetic kidney disease, while elevated circulating HNP1-3 levels correlate with markers of renal injury such as albuminuria and reduced eGFR. DEFA5, although predominantly expressed in Paneth cells, can enter the circulation under conditions of intestinal barrier dysfunction or systemic inflammation, potentially explaining its more modest associations and the borderline adjusted signal for nephropathy. DEFA3 demonstrated moderate discriminatory ability for diabetic nephropathy (AUC = 0.641; *p* = 0.037), superior to DEFA5 (AUC = 0.570; *p* = 0.303). After multivariable adjustment and correction for multiple comparisons, most associations were attenuated. This could indicate that DEFA3 could serve as a more sensitive surrogate of cumulative innate immune activation, whereas the attenuation after covariate adjustment indicates that both defensins largely represent downstream effectors rather than primary drivers of complications [22,24].

Strengths of this study include the well-characterized cohort with standardized, gold-standard assessments of multiple diabetic complications (KDIGO-based criteria for nephropathy, NDS for peripheral neuropathy and Ewing tests plus HRV for cardiac autonomic neuropathy), simultaneous measurement of both DEFA3 and DEFA5 using validated ELISA kits, comprehensive adjustment for key confounders, and application of the Benjamini–Hochberg false discovery rate correction for multiple testing. Limitations must also be acknowledged. The cross-sectional design precludes causal inference regarding whether defensin elevations precede or result from diabetic nephropathy. The modest sample size (n = 160 overall; 93 with T2DM) limits statistical power for subgroup analyses and may explain why the adjusted association for DEFA5 with nephropathy remained borderline. Residual confounding by unmeasured factors (e.g., diet, physical activity, or specific anti-diabetic medications) cannot be entirely excluded. High measurement variability for DEFA5 was noted and sensitivity analyses were performed where appropriate, with results remaining consistent. Finally, circulating levels may not fully capture local tissue expression or paracrine actions of alpha-defensins within the kidney or vasculature.

Taken together, these data position DEFA3 and, to a lesser extent, DEFA5 as biomarkers of innate immune dysregulation in diabetic nephropathy. They reinforce the emerging paradigm that neutrophil-driven inflammation and defensin release contribute to the progression of microvascular complications in T2DM. Future longitudinal studies in larger, multi-center cohorts are warranted to establish the temporal relationship between alpha-defensin dysregulation and the onset or progression of diabetic kidney disease, to evaluate their incremental value beyond traditional markers (eGFR and ACR), and to explore their potential as therapeutic targets or monitors of response to anti-inflammatory or renoprotective interventions.

## 5. Conclusions

In this monocentric cross-sectional study, circulating levels of the alpha-defensins DEFA3 (HNP-3) and DEFA5 (HD-5) did not differ significantly between patients with type 2 diabetes mellitus and non-diabetic controls. However, both peptides were markedly elevated in individuals with diabetic nephropathy, with DEFA3 demonstrating superior discriminatory capacity (AUC = 0.641, *p* = 0.037) and stronger correlations with diabetes duration, adiposity, dyslipidemia, and renal function parameters compared with DEFA5. Although unadjusted analyses revealed nominal associations of DEFA3 with both diabetic neuropathy and nephropathy, these relationships were largely attenuated after multivariable adjustment for diabetes duration, HbA1c, and renal markers, suggesting that alpha-defensins primarily reflect downstream innate immune activation and renal stress rather than serving as independent causal drivers of microvascular complications.

These findings extend previous observations that neutrophil-derived alpha-defensins are upregulated in diabetic kidney disease and position DEFA3 as a more sensitive biomarker of cumulative innate immune dysregulation in type 2 diabetes. By highlighting the differential roles of DEFA3 and DEFA5 in the inflammatory milieu of diabetic nephropathy, the present study reinforces the central involvement of neutrophil-mediated inflammation in the pathogenesis of microvascular complications. Future longitudinal and larger-scale investigations are warranted to validate the clinical utility of DEFA3 as a non-invasive biomarker for risk stratification.

## Figures and Tables

**Table 1:** Anthropometric characteristics of the study groups.

	Group 1 Diabetes Mellitus	Group 2 Control Group
Age (g)	58.6 ± 8.2 *	51.84 ± 7.8
Weight (kg)	98.2 ± 17.9 *	81.3 ± 19.2
BMI (kg/m^2^)	34.9 ± 5.8 *	29.9 ± 6.3
Waist (cm)	112.3 ± 14.2 *	105.0 ± 10.9
WHR	0.98 ± 0.11 *	0.90 ± 0.08
WSR	0.76 ± 0.07 *	0.63 ± 0.05

* *p* < 0.05. WHR—waist-to-hip ratio. WSR—waist-to-stature ratio.

**Table 2:** Cardiovascular risk factors.

	Group 1 Diabetes Mellitus	Group 2 Control Group
Systolic BP	133.0 ± 16.3	128.11 ± 12.1
Diastolic BP	80.9 ± 9.01	81.0 ± 9.4
Arterial hypertension	91.4% *	52.8%
Cholesterol	5.3 ± 1.5	5.4 ± 1.1
LDL-cholesterol	3.0 ± 1.1	3.4 ± 0.9
HDL-cholesterol	1.2 ± 0.3 *	1.4 ± 0.3
Triglycerides	2.6 ± 2.7	1.7 ± 1.1
Dyslipidemia	80.4% *	63.9%
Smoking	39.8%	50%
Metabolic syndrome	92.2% *	50%

* *p* < 0.05.

**Table 3:** Association of DEFA3 and DEFA5 with various biochemical and metabolic markers. Correlations were performed on the entire cohort (n = 160), except for diabetes duration, which was analyzed only in diabetic patients (n = 93).

	DEFA3	DEFA5
Diabetes duration	0.290 * (*p* = 0.006)	−0.018 (*p* = 0.867)
Weight	0.186 * (*p* = 0.019)	0.091 (*p* = 0.255)
BMI	0.178 * (*p* = 0.024)	0.094 (*p* = 0.238)
Cholesterol	0.222 * (*p* = 0.013)	0.305 * (*p* = 0.001)
LDL	0.262 * (*p* = 0.003)	−0.02 (*p* = 0.801)
Creatinine	0.242 * (*p* = 0.006)	−0.007 (*p* = 0.933)
eGFR	−0.207 * (*p* = 0.018)	0.023 (*p* = 0.795)
Uric acid	0.298 * (*p* = 0.001)	0.056 (*p* = 0.543)

**Table 4:** Area under the curve.

Test Result Variable(s)	Area	Std. Error ^a^	Asymptotic Sig. ^b^	Asymptotic 95% Confidence Interval	
				Lower Bound	Upper Bound
DEFA3	0.641	0.059	0.037	0.526	0.757
DEFA5 pg/mL	0.570	0.080	0.303	0.414	0.726

The test result variable(s): DEFA5ngml has at least one tie between the positive actual state group and the negative actual state group. Statistics may be biased. ^a^ Under the non-parametric assumption. ^b^ Null hypothesis: true area = 0.5.

## Data Availability

The original contributions presented in this study are included in the article. Further inquiries can be directed to the corresponding author.

## Abbreviations

The following abbreviations are used in this manuscript:

ACR	Albumin-to-creatinine ratio
AGEs	Advances glycation end-products
AUC	Area under the curve
BMI	Body mass index
CAN	Cardiac autonomic neuropathy
CI	Confidence interval
CKD	Chronic kidney disease
DEFA3	Alpha-defensin 3
DEFA5	Alpha-defensin 5
DKD	Diabetic kidney disease
DPN	Diabetic peripheral neuropathy
eGFR	Estimated glomerural filtration rate
ELISA	Enzyme-linked immunosorbent assay
FDR	False discovery rate
HbA1c	Glycated hemoglobin
HDL	High-density lipoprotein
HNP-3	Human neutrophil peptide-3
HD-5	Human defensin-5
IDF	International Diabetes Federation
KDIGO	Kidney Disease: Improving Global Outcomes
NDS	Neuropathy Disability Score
NETs	Neutrophil Extracellular Traps
NYHA	New York Heart Association
OR	Odds ratio
ROC	Receiver operating characteristic
SD	Standard deviation
T2DM	Type 2 diabetes mellitus
WHR	Waist-to-hip ratio
WSR	Waist-to-stature ratio
